# Can We Identify the Active Ingredients of Behaviour Change Interventions for Coronary Heart Disease Patients? A Systematic Review and Meta-Analysis

**DOI:** 10.1371/journal.pone.0153271

**Published:** 2016-04-22

**Authors:** Laura Goodwin, Giovanni Ostuzzi, Nadia Khan, Matthew H. Hotopf, Rona Moss-Morris

**Affiliations:** 1 Department of Psychological Medicine, Institute of Psychiatry, Psychology & Neuroscience, King’s College London, London, United Kingdom; 2 Department of Public Health and Community Medicine, Section of Psychiatry, University of Verona, Verona, Italy; 3 South London and Maudsley NHS Foundation Trust, London, United Kingdom; 4 Health Psychology Section, Institute of Psychiatry, Psychology and Neuroscience, King’s College London, London, United Kingdom; Kurume University School of Medicine, JAPAN

## Abstract

**Background:**

The main behaviour change intervention available for coronary heart disease (CHD) patients is cardiac rehabilitation. There is little recognition of what the active ingredients of behavioural interventions for CHD might be. Using a behaviour change technique (BCT) framework to code existing interventions may help to identify this. The objectives of this systematic review are to determine the effectiveness of CHD behaviour change interventions and how this may be explained by BCT content and structure.

**Methods and Findings:**

A systematic search of Medline, EMBASE and PsycInfo electronic databases was conducted over a twelve year period (2003–2015) to identify studies which reported on behaviour change interventions for CHD patients. The content of the behaviour change interventions was coded using the Coventry Aberdeen and London—Refined (CALO-RE) taxonomy. Meta-regression analyses examined the BCT content as a predictor of mortality. Twenty two papers met the criteria for this review, reporting data on 16,766 participants. The most commonly included BCTs were providing information, and goal setting. There was a small but significant effect of the interventions on smoking (risk ratio (RR) = 0.89, 95% CI 0.81–0.97). The interventions did not reduce the risk of CHD events (RR = 0.86, 95% CI 0.68, 1.09), but significantly reduced the risk of mortality (RR = 0.82, 95% CI 0.69, 0.97). Sensitivity analyses did not find that any of the BCT variables predicted mortality and the number of BCTs included in an intervention was not associated with mortality (β = -0.02, 95% CI -0.06–0.03).

**Conclusions:**

Behaviour change interventions for CHD patients appear to have a positive impact on a number of outcomes. Using an existing BCT taxonomy to code the interventions helped us to understand which were the most commonly used techniques, providing information and goal setting, but not the active components of these complex interventions.

## Introduction

Coronary heart disease (CHD) is the leading cause of death worldwide, for both communicable and non-communicable diseases [1]. In the UK, CHD remains a stable killer with approximately 74,000 deaths per year [2]. Within high-income countries there are more CHD deaths in areas of greater deprivation [3] and in individuals in manual occupational groups [4].

There is a widespread public health campaign promoting healthy behaviour, such as physical activity, which is based upon primary prevention literature about risk factors for CHD [5]. However, much of the secondary prevention guidance, such as the Quality and Outcomes Framework [6], focuses on blood pressure and cholesterol control, which can also be achieved through pharmacological intervention. This may be due to a mixed evidence base regarding individual health behaviours in secondary prevention; for example a systematic review of interventions involving reduced fat diets did not find a significant reduction in the risk of future CHD events or mortality [7].

For those CHD patients who want to try and change behaviours, the main lifestyle intervention available in Europe is cardiac rehabilitation, which primarily comprises education on CHD, disease and stress management, and physical activity classes. Such interventions are not based on theories of behaviour change, and UK evidence suggests that the benefits may be limited [8]. European data from EUROASPIRE-IV also suggests that a large proportion of CHD patients are not meeting clinical targets, for example 60% reported little or no exercise [9]. Furthermore, only half of patients were referred to a cardiac rehabilitation programme and not all of these actually attended [9]. Issues of non-attendance and adherence may be more pronounced in those groups who are at greatest risk, for example, individuals with lower socio economic status [10].

Effective behaviour change requires more than simply providing information on what changes need be made. Michie and colleagues have stressed the importance of behaviour change interventions being placed in the context of psychological theory and developed a taxonomy to classify such interventions, called the CALO-RE taxonomy [11]. Behaviour change taxonomies can be helpful both for those describing an intervention they are administering, and for classification of existing interventions for review, using a comprehensive coding system.

In addition to the known issues regarding definition of intervention content, addressed through frameworks such as the TIDieR checklist [12], there are also problems with evaluation. This most commonly relates to issues with the primary outcomes selected, which is then reflected in the selection of outcomes for inclusion in previous meta-analyses. If interventions are aiming to target health behaviours, then the meta-analyses should reflect this. Existing meta-analyses of heterogeneous pools of RCTS suggest that psychosocial interventions for CHD may be effective(e.g. [13]), finding a weighted relative risk of 0.82 for mortality. However what these reviews cannot tell us is what components of these interventions may be effective and the effect of these interventions on other outcomes such as health behaviours.

The objectives of this systematic review are to identify psychosocial or lifestyle behaviour change RCTs for CHD patients and to: i) determine the effect on health behaviours, intermediate outcomes of blood pressure and BMI, and CHD events and mortality and ii) to code the content of these interventions using the CALO-RE behaviour change taxonomy [11] and examine how the content and structure (length, format, theoretical basis) can predict effectiveness.

## Methods

This review was reported in accordance with PRISMA guidelines (see S1 Table).

### Search Strategy

The search for appropriate literature was conducted using the OvidSP search engine in February 2016, searching Medline, EMBASE and PsycInfo electronic databases. The search aimed to identify studies which reported on lifestyle or psychosocial behaviour change intervention programmes for patients with CHD. The search included studies over a twelve year period from 2003–2015 to capture more recent developments in behaviour change theory and intervention [14] and was restricted to articles that were published in the English language.

A search of keywords, abstracts, and titles was conducted for the following search terms: (*Intervention* or *rehabilitation* or *modification* or *prevention* or *promotion* or *management* or *programme* or *feedback*) combined using the AND command with (*behaviour* or *lifestyle*), AND (*myocardial infarction* or *coronary heart disease* or *coronary artery disease* or *heart attack* or *cardiac arrest* or *coronary infarction* or *cardiac infarction*) AND (*post* or *secondary*).

After having performed the search, titles and abstracts were downloaded into an electronic database and duplicates were excluded. Two review authors (LG and NK) independently assessed the eligibility of each study. Disagreements were discussed with a third author (GO).

### Inclusion criteria

The following inclusion criteria were applied to the articles:

1. Randomised controlled trial design, with no restriction on the length of follow-up.
2. Participants of 18 years of age or above, with a primary diagnosis of CHD, which included patients with unstable angina, patients who had undergone percutaneous coronary intervention (PCI) or coronary artery bypass graft (CABG), patients with acute coronary syndrome (ACS) or those who had suffered a myocardial infarction (MI).
3. At least 100 participants in order to exclude very small randomised trials or pilots.
4. Interventions should be psychosocial or lifestyle behaviour change interventions.
5. Must report on quantitative analyses.
6. Must report data on health behaviour outcomes, i.e. smoking, physical activity etc., although this did not have to be the primary outcome.
7. Peer reviewed research study.
8. Article written in the English language.

### Data extraction and analysis

Data was independently extracted by two researchers (NK and GO) and data extraction was conducted by both researchers for all articles. Inter-rater agreement was assessed by a third researcher (LG) who checked through the full extraction, and any queries were discussed between all three researchers. The data extracted from the articles included: authors, date, country, population description, sample size and characteristics, type of randomisation, intervention overview and content, length, number of sessions, who administered by, timing and number of follow-ups, description of control arm, theoretical basis and a description of the primary outcome. The outcome data which was extracted included: health behaviours (smoking, physical activity, diet, medication adherence), intermediate outcomes (body mass index, blood cholesterol, blood pressure), cardiovascular events, mortality and total drop-outs. Further details on the results were extracted from the papers which is available from the authors on request.

Authors were contacted to request additional information for the meta-analysis when it was not reported in the paper. These requests were generally for continuous outcome data at follow-up for intermediate outcomes, when either change scores or the proportion of the sample meeting a specified clinical criterion was reported.

### Content of the intervention

The content of the behaviour change interventions was coded using the Coventry Aberdeen and London—Refined (CALO-RE) taxonomy, which includes 40 behaviour change techniques [11]. This taxonomy includes a description of each of the techniques, with specific examples provided. Although there are items which appear similar, the taxonomy has been developed to avoid overlap; for example, technique 5 is ‘goal setting (behaviour)’ which is distinguished from technique 6 ‘goal setting (outcome)’, which relates to measurable outcomes such as blood pressure or weight loss. The BCT content of each intervention was rated by 3 researchers (NK, GO & LG) and any discrepancies were discussed and the final rating was agreed.

### Assessment of study quality

The methodological quality of the included studies was assessed with the “Cochrane Risk of Bias Tool” [15], which aims to evaluate the risk for the most relevant biases, assigning a judgement of “low”, “unclear” or “high risk” for six different domains. These criteria included assessment of the internal validity of the trial and the quality of reporting Two authors (GO, LG) independently assessed the quality of each study. Disagreements were discussed and, if necessary, a third author (MHH) was consulted.

### Statistical analysis

Stata v11.0 was used for all data analyses [16] and meta analyses were conducted to produce weighted estimates and to examine the between study heterogeneity. For continuous outcomes, the weighted mean difference estimates, indicating the difference in means between the intervention and control group, were computed using the sample size, mean and standard deviation (SD) at follow-up for the intervention and control groups. For categorical outcomes, the risk ratio was computed based upon the number of cases (i.e. events) and the number of non-cases (i.e. non-events) during the study period for both the intervention and control groups. For smoking, the outcome data was reported differently between studies. For the purposes of this review, the proportion of smokers and non-smokers at follow-up was calculated for all studies. The *I*^*2*^ statistic was used to assess between study heterogeneity [17]. Random effects models were conducted as it was predicted that there would be between study heterogeneity resulting from differences in the populations and the interventions. The meta-analyses for both mortality and smoking were also stratified by i) study length, ii) whether they were individual or group interventions, and iii) if the intervention had a theoretical basis for the outcomes. Sensitivity analyses were conducted examining the BCT content of the interventions as a predictor of mortality in meta-regressions. The most commonly applied BCTs were analysed in the meta-regression using the following categories: provide information—BCTs 1, 2, 20 & 21; goal setting/action planning—BCTs 5, 6 & 7; review of goals/self-monitoring—BCTs 10, 11, 16 & 17; stress management—BCT 36; social support—BCT 29, and provide feedback—BCT 19.

## Results

### Study selection

**Fig 1:**
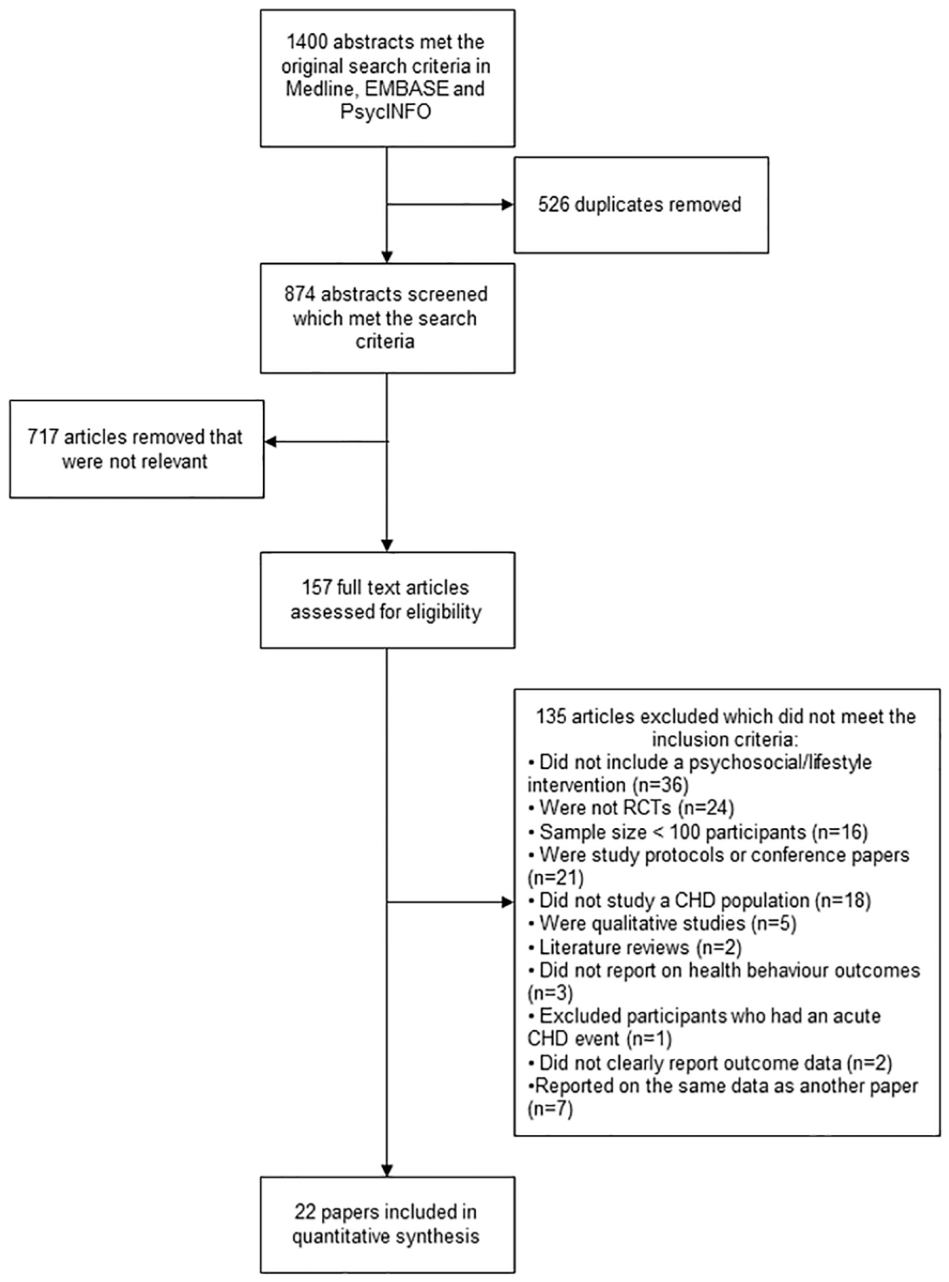
PRISMA flow diagram showing the search process and selection of relevant abstracts.

The original search revealed 1400 articles, 874 of which remained after duplicates had been removed and 157 remained after irrelevant articles were removed (see Fig 1). After full text review, twenty two papers met the inclusion criteria and were included in this review [8, 18–37] which in total reported data on 16,766 participants.

### Overview of the interventions

See Table 1 for full details of the intervention studies. The intervention length differed between studies; five interventions lasted less than 3 months [8, 23, 25, 29, 38], ten were 3–6 months in length [18, 21, 22, 24, 30, 33–37], four lasted for 12 months [19, 27, 31, 32], one was 18 months [28] and two lasted as long as 3 years [20, 26]. The sample sizes ranged from 120 for the smallest study [35] to 3241 participants in the largest [20].

**Table 1:** Data extraction table showing methods of RCT and overview of intervention.

Study information					Overview of the intervention								
Authors	Population	Sample size	Sample characteristics	Type of randomisation	Intervention content	Length	CALO-RE techniques	Delivery & no. of sessions	Intervention administered by	Timing and number of follow-ups	Control condition	Intervention fidelity	Theoretical basis to intervention
Berndt et al., 2013, Netherlands	Patients who had been hospitalised for acute coronary syndrome (ACS), stable angina, or other chronic and acute heart diseases. Must smoke on average > = 5 cigarettes a week, or quit < 4 weeks prior to admission.	625 (IG 1 (TC): 157, IG 2 (FC): 223, CG: 245)	IG 1: 223, 163 males (73.1%), mean age 55.3 (sd 10.5). IG 2: 157, 111 males (70.7%), mean age 56.5 (sd 10.5). CG: 245, 183 males (74.7%), mean age 56.1 (sd 11.0).	Sequential cross-over randomisation at the ward level. After completion of care as usual the cardiac wards were randomised to implement either TC or FC, and then after completion and a month wash-out period implemented the other intervention.	TC (IG 1): Nurses on cardiac wards followed the Ask-Advice-Refer strategy prior to the counselling sessions. The counsellors worked with a protocol based upon the Transtheoretical Model and within each session they discussed relevant themes and focused on determinants of smoking cessation and relapse information important for each stage. FC (IG 2): The content and structure of FC was highly comparable to TC other than TC was delivered by professional telephone counsellors.	3 months	IG 1 & 2: 1, 2, 5, 6, 10, 19, 21, 35. Additional smoking cessation taxonomies: BM1,BM2,BM3,BM9,BS1,BS2,BS4,BS5,A1,RI1,RC5	Individual. IG 1: 7 telephone sessions of 10 to 15 min. IG 2: 6 face-to-face sessions of 45 min and a follow-up call eight weeks after the last session.	Smoking cessation counsellors who were nurses	6-month telephone follow-up	Standard in-hospital treatment for smoking cessation which consisted of an assessment of smoking behaviour and personalised brief quit advice.	No formal evaluation of intervention fidelity	The Transtheoretical Model (TTM)
Blasco et al., 2012, Spain	Patients with ACS with at least 1 risk factor: (1) tobacco smoking, (2) LDL-c > = 100 mg/dL, (3) hypertension, or (4) diabetes mellitus.	203 (IG: 102, CG: 101)	IG: 81.4% male, mean age 60.6 y (sd 11.5); CG: 79.2% male; mean age 61.0 (sd 12.1)	Single blind randomisation, stratified by DM status	Telemedicine intervention including monitoring of clinical outcomes (e.g. using sphygmomanometer) and patients sent their results through their mobile phone to a cardiologist.	12 months	6, 11, 17, 21	Individual. Weekly telemedicine text messages and 3 clinical visits	Cardiologist	Follow-up at 12 months only	All patients received lifestyle counselling and usual care treatment	Adherence to protocol was measured by the percentage of WAP sessions completed. 98% of patients completed more than 50% of WAP sessions and 83% completed more than 75%. Only 0.5 messages per patient were missed, due to the mobile phone being turned off.	No
Bond et al. 2007, England	Patients aged over 17 years, with CHD (previous myocardial infarction, angina, coronary artery bypass graft and/or angioplasty)	1493 (Data collected from 1441, IG: 941, CG: 500)	IG: 941, 634 males (67.4%), mean age 68.7 (sd 9.2). CG: 500, 353 males (70.6%), mean age 68.8 (sd 9.1).	Patients were randomised independently of the research team, using a computer programme in permuted blocks stratified by practice	The intervention was delivered by community pharmacists. Consultations included assessments of the following: therapy, medication compliance, lifestyle and social support.	12 months	1,2,19,29	Individual. No. of sessions not reported.	Trained pharmacists	12 months from the date of the first pharmacy appointment	Usual care	No formal evaluation of intervention fidelity	No
Chow et al., 2015, Australia	Patients with CHD (MI, CABG, PCI or > = 50% stenosis) recruited at a large teaching hospital in Sydney, Australia	710 (IG: 352, CG: 358)	IG: 81.5% male, mean age 57.9 (S.D. 9.1); CG: 82.4% male, mean age 57.3 (S.D. 9.3)	Computerised randomisation in a uniform 1:1 allocation ratio with a block size of 8, concealed from study personnel	Text message based intervention involving semi-personalised messages, providing advice, motivation and information about lifestyle. Content of messages based upon baseline characteristics.	6 months	Outlined in protocol. 1, 2, 5, 8, 9, 12, 17, 21, 23, 35, 38	Individual. 4 text per week for 6 months.	Automated text messages based upon a pre-specified algorithm.	6 months only	Usual care, which included community follow-up and referral to inpatient cardiac rehabilitation.	Logs of the number of messages delivered and responded to were kept. 87% completed a feedback questionnaire on the utility and acceptability of the programme.	Based upon a range of theories including control theory, information-motivation-behavioural skills model and theory of planned behaviour.
Dale et al., 2015, New Zealand	Patients were English speaking adults with CHD (MI, angina or revascularisation) recruited from 2 hospitals in Auckland, New Zealand. Patients required to have access to the internet.	123 (IG: 61, CG: 62)	IG: 79% male, mean age 59.0 (S.D. 10.5), CG: 84% male, mean age 59.9 (S.D. 11.8).	One-to-one randomisation, stratified according to smoking status. Randomisation sequence was computer generated by a statistician independent to project using block size of 6	Comprehensive programme of evidence based CR guidelines delivered by text message and a supporting website, providing education about CV risk factors and supporting patients to make lifestyle changes.	24 weeks	1, 2, 4, 5, 6, 7, 8, 9, 10, 11, 12, 13, 19, 20, 24, 26	Individual. 7 messages per week, reduced to 5 per week from wks 13–24.	Text messaging	3 and 6 months	Usual care which included inpatient rehabilitation and encouragement to attend centre based CR	Fidelity was assessed using an author-derived questionnaire and through calculating website and response text message usage statistics. 85% of participants reported reading all of their texts. 75% logged onto website at least once.	Social cognitive theory and Common Sense Model
Giannuzzi et al., 2008, Italy	Patients with a recent MI (within 3 months) irrespective of revascularization procedures received after the index event.	3241 (IG: 1620, CG: 1621)	IG: 85.9% male; mean age 57.8 (sd 9.1); CG: 86.7% male; mean age 58.0 (sd 9.3)	Open label randomisation after the standard 1-month cardiac rehabilitation programme	A multifactorial, continued education and behavioural cardiac programme including cardiac rehabilitation, and meetings with family members.	3 years	1,2,5,6,10,11,20,21,22,29,35	Group. Monthly from month 1 to 6, then every 6 months for 3 years	Cardiac rehabilitation team (specialist cardiac nurse, physiotherapist, cardiologist).	Follow-up visits 6 months, 1, 2, 3 years, and then yearly (minimum 3 years)	Usual care (which included the 1-month rehabilitation programme) and a letter to GP recommending secondary prevention goals	Intervention fidelity was not reported as the intervention included 78 cardiac rehabilitation programmes	No
Hanssen et al., 2007, Norway	All patients with an AMI confirmed through medical records, and admitted to the hospital	288 (IG: 156, CG: 132)	IG: 84.6% male; mean age 59.5y (sd 12.9). CG: 76.5% male; 60.9y (sd 10.8)	Simple randomisation using computer generated list of random number	Nurse led telephone follow-up intervention to provide information and support to patients after their discharge from hospital.	6 months	1,2,5,6, 8, 20, 21, 35, 36	Individual. 8 phone calls in 6 months (average 6.9 mins)	Nurse	3 and 6 months	Current clinical practice—one visit to a physician at the outpatient clinic and subsequent visits to GP	No formal evaluation of intervention fidelity	Lazarus and Folkman’s theory of stress, appraisal, and coping
Hawkes et al., 2013, Australia	Eligibility criteria included a diagnosis of MI or coronary artery intervention, ages 18–80 years	430 (IG: 215, CG: 215)	IG: 215, 163 males (75.8%), mean age 61.3 (sd 11.3). CG: 215, 158 males (73.5%), mean age 59.9 (sd 11.1).	Participants were randomised to the intervention or control group following enrolment	The health coaching (HC) telephone intervention focused on the core determinants of health behaviour including knowledge of the risks and benefits of the behaviour, self-efficacy or confidence that one can engage in the behaviour under various circumstances, outcome expectations and individualised strategies for achieving positive health behaviour change.	6 months	1,2,5,6,8,10,11,19,20,21,27,29,36	Individual. 10×30 minute telephone calls	Health coaches	6-months	UC participants received the educational resource ‘My Heart My Life’ and quarterly informative to enhance participant retention.	The intervention protocol was manualised, and all intervention calls were audio-taped with 10% reviewed against a session checklist. The sessions were also reviewed by a second rater to investigate inter-rater reliability, with 98% agreement between reviewers. The health coaches met with study investigators for bi-weekly supervision sessions.	Social cognitive theory
Jolly et al., 2007, UK	Patients who had experienced an MI or coronary revascularisation (PTCA/CABG) within the previous 12 weeks	525 (IG (Home-based): 263, CG: 262)	IG: 77.2% male; mean age 60.3 (sd 10.5). CG: 75.9% male; mean age 61.8 (sd 11.0)	Randomisation on an individual basis with minimisation by diagnosis, age, sex, ethnicity and hospital of recruitment, using a customised computer program	Home based cardiac rehabilitation programme, comprising a manual, home visits and telephone contact.	6 weeks	1,2,5,6,20,21,22,36	Individual. Daily home based sessions	Nurse	6, 12 and 24 months	Hospital-based cardiac rehabilitation which differed by hospital.	Assessed participant adherence to the programmes, but not fidelity of the programme delivery	Health Belief Model
Jorstad et al., 2013, The Netherlands	Participants with an acute coronary syndrome within 8 weeks prior to entry into study.	754 (IG: 375, CG: 379)	Received the intervention: G: 366, 293 males (80%), mean age 57.5 (sd 9.9). CG: 367, 293 males (80%), mean age 57.8 (sd 10.4).	Block-stratified randomisation	A nurse-coordinated prevention programme which followed a protocol based on national and international guidelines.	6 months	1,2,5,6,20,21,22	Individual. 4 outpatient visits (at week 2, 7, 12, and 17 after baseline)	Cardiovascular nurses	6 and 12 months	Outpatient clinic visits to cardiologists and referral to cardiovascular rehabilitation according to national guidelines	Individual nurses were observed on at least two separate occasions by study personnel. Video recordings were also made of the nurses’ consultations that were evaluated by a medical psychologist, who provided feedback to the nurses.	No
Melamed et al., 2014, Germany	Patients with CHD aged 18–89 years recruited by primary care physicians and cardiologists in Frankfurt	395 (IG: 196, CG: 199)	IG: 79.1% male, mean age 65.7, CG: 79.4% male, mean age 65.8	Randomisation conducted at the central coordinating centre and reported immediately to the study practices	Educational programme delivered across 5 primary care practices involving a patient brochure, independent study, teaching cards and an exercise diary.	6 months	1, 2, 16, 21	Group. 3 sessions at time intervals of 7 days.	Physicians and medical assistants	6 months only	Usual care from primary care physician/cardiologist	No formal evaluation of intervention fidelity.	No
Muniz et al., 2010, Spain	Patients with acute coronary syndrome, discharged with a diagnosis of Q-wave or non-Q-wave acute MI or unstable angina	1,757 (IG: 867, CG: 890)	IG: 77.7% male, mean age 62.1 (s.d.11.6), CG: 75.6% male, mean age 63.6 (s.d. 11.4)	Open label randomisation by individual and stratification by centre	The intervention consisted of a signed agreement between patient and physician on the specific secondary prevention procedures and the therapeutic aims.	2 months	1, 5, 6, 7, 10, 11, 21, 25, 29	Individual. 2 sessions each lasting 30/40 minutes	Physician	6 months	Usual care	No formal evaluation of intervention fidelity	No
Munoz et al., 2007, Spain	Patients aged 30–79 years who had suffered MI or angina with electrocardiographic signs of ischaemia in the 6 years prior to recruitment	983 (IG: 515, CG: 468)	IG: 515, 392 males (76.1%), mean age 64.2 (sd 9.8). CG: 468, 343 males (73.2%), mean age 63.6 (sd 10.3).	Primary care health centres were randomly allocated using a random sequence generated by a computer programme	GPs in the intervention centres were instructed to follow the most recent guidelines on cardiovascular prevention and received a copy of the study protocol which included detailed recommendations and outlined the treatment objectives.	3 years	1,2,19,20,21,	Individual. Participants received a quarterly reminder to meet with their GP	GPs	3 years or until an end-point occurred	Usual care	GP adherence to the protocol in the intervention group was monitored by quarterly reporting	No
Murchie et al., 2003, UK	Patients with a working diagnosis of coronary heart disease, but without terminal illness or dementia and not housebound	1343 (IG: 673, CG: 670)	IG: 58.2% male, mean age 66.1 (sd 8.2). CG: 58.2% male, mean age 66.3 (sd 8.2)	Randomisation by individual stratified by age, sex and practice using tables of random numbers	Nurse led secondary prevention clinics in general practice. Each clinic visit ended with feedback, goal planning, and an agreed action plan.	1 year	1,2,5,6,10,11, 19	Individual. Every 2–6 months. First visit 45 minutes and follow-ups approx. 20 minutes	Nurse	1 year and 4 years	Usual care by the GP	No formal evaluation of intervention fidelity and individual clinics could amend their protocols	No
Murphy et al., 2009, Northern Ireland and Republic of Ireland	Patients with established coronary heart disease. Patients with a major mental or physical illness were excluded.	903 (IG: 444, CG: 459)	IG: 444, 311 males (70%), mean age 68.5 (sd 9.3). CG: 459, 320 males (70%); mean age 66.5 (sd 9.9).	Cluster randomisation. Practices were stratified according to numbers of whole time equivalent GPs.	Tailored care plans for practices (including practice based training in drug prescribing guidelines and behaviour change). Tailored care plans for patients (including motivational interviewing, goal identification, and goal setting for lifestyle change) with reviews every four months at the practices.	18 months	1,2,5,6,10,11,20,21,37	Individual. Every four months	GPs and nurses	Every 4 months. Last assessment at 18 months	Usual care	No formal evaluation of intervention fidelity	Social cognitive theory
Murphy et al., 2013, Australia	Patients admitted to hospital after an AMI or to undergo a coronary artery bypass graft surgery (CABGS) or a percutaneous coronary intervention (PCI) and < 75 years	275 (IG: 139, CG: 136)	IG: 139, 124 males (89.2%) mean age 58.02 (sd 8.87). CG: 136, 114 males (83.8%), mean age 59.92 (sd 9.27).	Randomisation occurred after the baseline risk factor screening to ensure that the nurse was blind to allocation. Patients were randomised on a 1:1 basis.	The “Beating Heart Problems” program is a face-to-face cognitive behavioural therapy (CBT) and motivational interviewing (MI) group programme. It includes modules on physical activity, diet, medication adherence, smoking cessation, depression, anxiety, anger, and social support.	8 weeks	1,2,5,6,8,9,10,24,29,35,36,37	Group. 8 weekly sessions of 1.5 hours each	Registered psychologists and nurses	4 and 12 months	Usual care and attendance at cardiac rehabilitation was monitored	Treatment fidelity was not formally assessed. To ensure treatment fidelity, the program developers facilitated the sessions and supervised the 2 co-facilitators. A practitioner manual was used and all materials were piloted before commencement of the trial.	Cognitive behavioural therapy and motivational interviewing
Otterstad et al., The Vestfold Heartcare Study Group, 2003, Norway	Patients with AMI, unstable angina pectoris, percutaneous coronary intervention, coronary artery bypass grafting.	197 (IG: 98, CG: 99)	IG: 81% male, mean age 54 (sd 8.0). CG: 84% male, mean age 55 (sd 8.0)	Patients randomised using pre-prepared sealed opaque envelopes including information on group allocation. Patients opened the envelopes themselves so study investigators were blind to allocation.	Six-week period of "heart school": a multidisciplinary cardiac rehabilitation and lifestyle intervention.	6 weeks 'Heart School' + 9 weeks organised physical exercise	1,2,20,21,22,29,36	Group. Heart school lasted 6 weeks & the 9-week exercise programme was twice weekly	The study physician ran the heart school with two study nurses, a physiotherapist and a clinical nutritionist.	Six months and 2 years	Usual care and standardised information on CHD and lifestyle measures	No formal evaluation of intervention fidelity	No
Varnfield et al., 2014, Australia	Post MI patients referred to CR in Queensland, Australia. Patients were required to be able to participate in a self-management programme and to use a smartphone.	120 (IG: 60, CG: 60)	IG: 91% male, mean age 54.9 (S.D. 9.6), CG: 83% male, mean age 56.2 (S.D. 10.1)	Permuted-block randomisation, by computer generated random numbers with variable block sizes (4, 6 & 8), using sequentially numbered opaque, sealed envelopes	Smartphone intervention for health and exercise monitoring (e.g. health diary, step counter) and delivery of motivational and educational materials via text messages and preinstalled audio/video files.	6 months	1, 2, 5, 6, 10, 13, 16, 17, 19	Individual. Self monitoring through smartphone and weekly telephone consultation with mentor for 6 weeks.	Smartphone app and mentor	6 weeks and 6 months	Traditional centre based CR programme comprising 2 exercise sessions and 1 hr education per week for 6 weeks	Assessed through smartphone physical activity data and questionnaire. Questionnaire indicated that > 85% found the step counter to be motivational in reaching CR goals.	No
West et al., 2012, England and Wales	Admission to hospital with a principal primary diagnosis of acute MI (two of the three standard criteria ‘typical history’, electrocardiographic features and cardiac enzymes).	1,813 (IG: 903, CG: 910)	IG:72.6% male, mean age 64.2 (sd 11.2). CG: 74.4% male, mean age 64.7 (sd 10.9).	Patients were randomised centrally on a pre-set protocol, blind as to entry characteristics and baseline measures.	Rehabilitation programmes comprised exercise training, health education on heart disease, risk factors and treatment, counselling for recovery and advice for long-term secondary prevention.	6–8 weeks (depending on the centre)	1,2,20,21,22,35,36	Group. Weekly or bi-weekly and averaged 20 h over 6–8 weeks	Nurses with previous acute cardiac care experience, occupational therapists or physiotherapists.	1 and 2 years. Mortality after 7–9 years was traced at the NHS central registry.	Usual care	No formal evaluation of fidelity of the different CR programmes	No
Wister et al., 2007, Canada	Patients aged 45–64 years with coronary artery disease (only the secondary prevention group included)	296 (IG: 153, CG: 143)	IG: 66% male, mean age 56.6 (sd 5.1). CG: 72% male, mean age 57.2 (sd 5.0).	Randomisation by individual stratified by smoking status using computer generated random numbers. Outcome assessors were blinded to group allocation.	The intervention consisted of a report card showing the person’s risk profile, coupled with a Telehealth-guided self-care management system.	1 year	1,2,5,6,10,11,20,21, 36	Individual. One 30 min. session every 6 months. Additional sessions for smokers at 2, 4, 8 and 12 weeks.	Clinical lifestyle counsellors (kinesiologists)	1 year	Usual care	No formal evaluation of intervention fidelity	No
Yan et al., 2014, China	Patients who presented with an initial MI to cardiac care units in Guangzhou (Southern China) who could communicate orally in Mandarin or Cantonese and read in Chinese	124 (IG: 62, CG: 62)	IG: 78.4% male, mean age 64.25 (S.D. 11.72), CG: 72.5% male, mean age 64.29 (S.D. 12.77)	Randomisation took place after completion of the baseline questionnaire. The randomisation sequence was generated using a computerised random number generator and the allocation was kept in sealed consecutively numbered envelopes.	Intervention based on the Self-Regulation Theory involving a pre-discharge education session and three telephone follow-ups to discuss illness beliefs and lifestyle. Patients were provided with an educational handbook.	12 weeks	1, 5, 12, 19	Individual. One face-to-face session, 3 telephone sessions.	Research assistant.	6 and 12 weeks	Usual care	Fidelity not directly discussed but the research assistants received intensive training and supervision in the delivery of the intervention.	Self regulation theory
Zhao et al., 2008, China	Patients at least 60 years old, with a confirmed diagnosis of anginaor MI, who would be able to be reached by telephone post-discharge	220 (IG: 107, CG: 113)	IG: 51% male, mean age 72.86 (sd 6.43). CG: 47% male, mean age 71.58 (sd 4.14). [Data for patients who completed the study.]	Patients were randomised using a computer-generated randomised table	The transitional care programme consisted of pre-discharge assessment, structured home visits and telephone follow-ups.	Pre-discharge and 4 weeks post-discharge	1,2,5,6,10,11,19,20,21	Individual. 3 face-to-face and 2 telephone calls	Specialist nurses	Before discharge, 2 days, 2 weeks, 4 weeks, and 12 weeks after discharge	Usual care (visits with the doctor & educational pamphlet)	The research team randomly chose 10% of the cases and reviewed the telephone calls to ensure that the intervention delivered complied with the protocol	No

The interventions were delivered most commonly by nurses (six of the studies)[8, 21, 23, 24, 27, 38], followed by counsellors or coaches in 4 studies [18, 22, 32, 36], by doctors in 3 studies [19, 25, 26, 37], two were delivered by a cardiac rehabilitation team [20, 30], three through automated text messages [33–35] and the three remaining studies were delivered by pharmacists [31], by both GPs and nurses [28], and finally by both psychologists and nurses [29]. Seventeen of the interventions were delivered on an individual basis, either in person [23–28, 31, 38] or over the telephone [18, 19, 21, 22, 32–36], and five were group interventions [8, 20, 29, 30, 37].

The control condition differed between studies, but most commonly comprised “usual care”, which in seven studies involved a cardiac rehabilitation programme [20, 23, 24, 29, 33–35]. Only nine of the seventeen studies reported that they had a theoretical basis [18, 21–23, 28, 29, 33, 34, 36]. The primary outcome differed between studies. Four studies reported that the primary outcome was a derived CHD risk score or algorithm [24, 29, 30, 32], with a further five reporting multiple primary outcomes [22, 23, 27, 37, 38]. Four studies defined the primary as achieving a range of clinical targets [19, 25, 28, 34].

### Inclusion of BCTs

**Fig 2:**
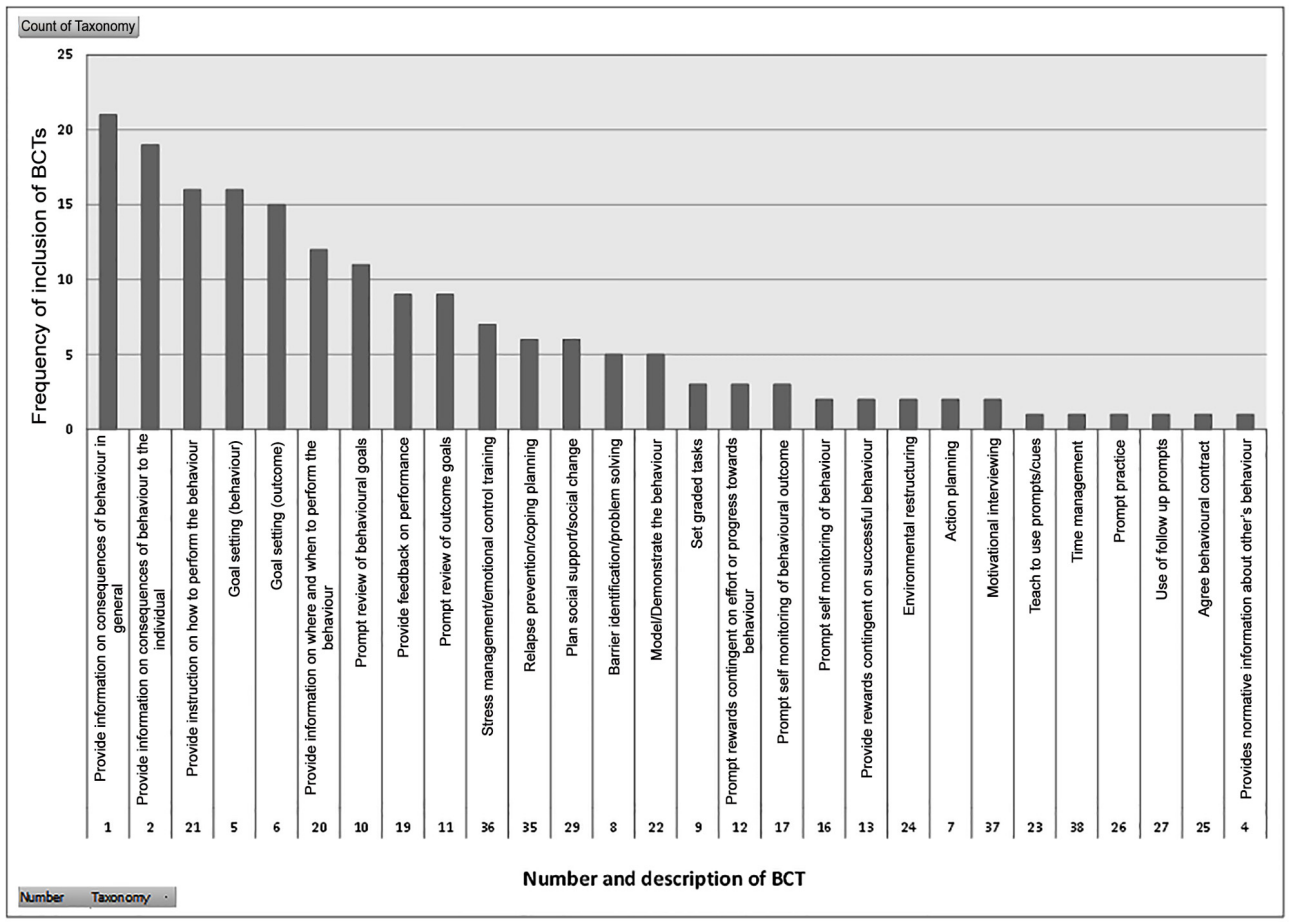
Frequency of inclusion of behaviour change techniques.

Fig 2 displays the frequency of inclusion of the different BCTs. The most commonly included techniques were providing information on the consequences of behaviour (BCTs 1 &2), providing instruction on how to perform the behaviour (BCT 21), goal setting in relation to the outcome (BCT 6) and the behaviour (BCT 5), providing information on where and when to perform the behaviour (BCT 20) and prompt review of behavioural goals (BCT 10). Review of outcome goals (BCT 11) was less commonly reported. The mean number of BCTs included in the interventions was 8.3 (SD 3.1), ranging from 4 to 16 BCTs.

### Effectiveness of the interventions (see Table 2)

**Table 2:** Results for primary outcome and overview of additional findings.

Results of the RCT											
Authors	Definition of the primary outcome	Primary outcome (if reported)	Smoking	Physical activity	Diet	Medication adherence	BMI	Blood cholesterol/ lipids	Blood pressure	Coronary and cardiovascular events	Mortality
Berndt et al., 2013, Netherlands	Continued abstinence of smoking defined as being abstinent for at least 90 days	Continued smoking abstinence: 6 months: IG 1: 42.2% vs IG 2: 40.6% vs CG: 31.5% (IG1 vs CG: p = 0.02, IG2 vs CG: p = 0.06. No sig. difference IG1 vs IG2: p = 0.76).	Favours intervention (IG1)	Not reported	Not reported	Not reported	Not reported	Not reported	Not reported	Not reported	IG1: 5 (2.24%), IG2: 2 (1.27%), CG: 10 (4.08%)
Blasco et al., 2012, Spain	“Cardiovascular risk improvement” defined as the proportion of patients who achieved the goal of treatment in at least 1 coronary risk factor without exacerbation of any of the others.	Improvement in CVD risk: IG 69.6% vs. CG 50.5% (RR = 1.4, 95%CI 1.1–1.7)	No difference	No difference	Not reported	No difference	Favours intervention	No difference	Favours intervention	Not reported	IG: 0 deaths, CG: 5 deaths
Bond et al. 2007, England	Proportion of participants receiving secondary prevention treatment for CHD in accordance with the National Service Framework, and health status (SF-36, EQ-5D)	Total score for appropriate treatment of CHD (point given for each treatment target achieved): 12 months: IG: 4.6 (S.D. 1.2) vs CG: 4.6 (S.D. 1.1), Mean diff. = 0.19 (-0.07–0.46) p = 0.15	No difference	No difference	No difference	No difference	No difference	No difference	No difference	Not reported	IG: 22, CG: 20
Chow et al., 2015, Australia	Level of plasma LDL-C at 6 months.	IG: mean 79 (95% CI 76–82), CG: mean 84 (95% CI 81–87). Mean difference: -5 (-9 to 0), p = 0.04.	Favours intervention	Favours intervention	Not reported	No difference	Favours intervention	Favours intervention	Favours intervention	Not reported	IG: 4 deaths, CG: 1 death.
Dale et al., 2015, New Zealand	Self-reported composite health behaviour score based on the European Prospective Investigation into Cancer (EPIC) Norfolk Population Study.	Categorised as adherent if they scored 3 out of 4 behaviours. 6 months: IG: 53%, CG: 39%, AOR = 1.95, 95% CI 0.83–4.53, p = 0.13.	No difference	No difference	Favours intervention	Favours intervention	No difference	No difference	No difference	Not reported	Not reported
Giannuzzi et al., 2008, Italy	Combined endpoint included cardiovascular mortality; non fatal MI; non fatal stroke; hospitalisation for heart failure and angina pectoris; and urgent unplanned revascularisation procedure	IG: 16.1% vs CG: 18.2%. HR 0.88 (0.74–1.04) (p = 0.12) (% reports on occurrence of any of the events)	Favours intervention at 6 months, no difference at 3 yrs	Favours intervention	Favours intervention	Not reported	No difference	Favours intervention	No difference	Favours intervention	CV mortality: 3 years: IG: 1.1% vs CG: 1.5%, HR 0.75 (0.41–1.38). Total mortality: 3 years: IG: 2.1% vs CG: 2.7%, HR 0.79 (0.50–1.23).
Hanssen et al., 2007, Norway	Health-related quality of life (HRQOL) at 6 months using the 36-item Short Form Health Survey	SF-36 Overall physical score. 6 months: Mean difference: -2.33 (95% CI -4.54, -0.12). SF-36 Overall mental score. 6 months: Mean difference: 1.07 (-1.71, 3.86)	No difference	Favours intervention	Not reported	Not reported	Not reported	Not reported	Not reported	Not reported	Deaths and serious adverse events. IG: 5, CG: 6
Hawkes et al., 2013, Australia	Primary outcome variables were QoL and physical activity	Sufficiently active (≥150 min/week): 6 months: IG: 55.1% vs CG: 44.1%, OR 1.7 (1.1, 2.7) (p = 0.02).	No difference	Favours intervention	No difference	Not reported	Favours intervention	Not reported	Not reported	Not reported	IG: 2, CG: 0
Jolly et al., 2007, UK	The primary outcomes were serum cholesterol, blood pressure, exercise capacity, psychological morbidity and Cotinine-validated smoking cessation. Outcomes were reported individually.	See individual columns	No difference	No difference	Favours intervention at 6 months, no difference at 24 months	No difference	Not reported	No difference	No difference	No difference	Total deaths: 6 months: IG: 3 vs CG: 2, 24 months: IG:6 vs CG: 3; Cardiac deaths: 6 months: IG: 3 (1.1%) vs CG: 2 (0.8%) (p = 1.0) 24 months: IG: 6 (2.3%) vs CG: 3 (1.1%) (p = 0.3)
Jorstad et al., 2013, The Netherlands	The Systematic Coronary Risk Evaluation (SCORE) at 12 months which estimates the 10 year risk of cardiovascular death based on age, gender, total cholesterol, systolic blood pressure and smoking status.	12 months: IG: 4.4% (sd 4.5) vs CG: 5.4% (sd 6.2) (p = 0.021). Absolute reduction of 0.93% for IG (p<0.001) and increase of 0.17 for CG (p = 0.38).	No difference	Favours intervention	Favours intervention	No difference	No difference	Favours intervention	SBP: Favours intervention. DBP: No difference.	Not reported	IG 3 (0.8%%); CG 10 (2.7%).
Melamed et al., 2014, Germany	Physical activity (MET/week) and Disease related quality of life	Physical activity: IG: mean 41.1 (S.D. 31.9), CG: 31.5 (S.D. 29.5), p = 0.015. QoL: IG: mean 5.75 (S.D. 0.87), CG: mean 5.74 (S.D. 0.83), p = 0.056.	No difference	Favours intervention	Not reported	Not reported	Not reported	Not reported	Not reported	No difference for inpatient treatment for CHD	Not reported
Muniz et al., 2010, Spain	Reaching therapeutic objectives: smoking cessation, BMI < 25, doing regular exercise, controlling lipid levels, controlling hypertension and taking prescribed medication. Outcomes were reported individually.	See individual columns	No difference	Favours intervention	Not reported	No difference	No difference	No difference	No difference	Not reported	IG: 17 (2%), CG: 22 (2.5%)
Munoz et al., 2007, Spain	Admission for unstable angina, MI, heart failure, arrhythmias, stroke or coronary artery revascularisation	All cardiac events: 3 years: IG: 103 (20.0%) vs CG: 88 (18.8%), HR 0.90 [0.56–1.45] (p = 0.76).	No difference	Not reported	Not reported	Not reported	No difference	No difference	Favours intervention	No difference	Cardiovascular mortality: 3 years: IG: 11 (2.1%) vs CG: 15 (3.2%), HR 0.95 [0.46–1.98] (p = 0.89). All-cause mortality: 3 years: IG: 25 (4.9%) vs CG: 34 (7.2%), HR 0.79 [0.47–1.34] (p = 0.38).
Murchie et al., 2003, UK	Secondary prevention, total mortality, and CHD events. Secondary prevention definition: aspirin taken, blood pressure managed (guidelines of the British Hypertension Society), lipids managed (guidelines for lipid management in GPs in Grampion region), moderate physical activity (index of physical activity >4), low fat diet, and not smoking. Outcomes were reported individually	Coronary death or nonfatal MI: IG 14.9% vs CG 18.7%, RR 0.80 (95% CI 0.63, 1.01). See individual columns for secondary prevention outcomes.	No difference	Favours intervention at 1 yr, no difference at 4 yrs.	Favours intervention at 1 yr, no difference at 4 yrs.	Favours intervention at 1 yr, no difference at 4 yrs.	Not reported	Favours intervention at 1 yr, no difference at 4 yrs.	Favours intervention at 1 yr, no difference at 4 yrs.	No difference	Total mortality: IG 14.9% vs CG 19.1%, RR 0.78 (95% CI 0.61, 0.99)
Murphy et al., 2009, Northern Ireland and Republic of Ireland	The main outcomes were the proportion of patients at 18 months above target levels for blood pressure and total cholesterol concentration; hospital admissions; and changes in physical and mental health status (SF-12). Outcomes were reported individually	SF-12 mental component: 18 months: IG: 49.6 (s.d. 10.9) vs CG: 48.9 (s.d. 11.7). Mean difference −0.02 (−2.40 to 2.35) p = 0.98. SF-12 physical component: 18 months: IG: 40.5 (s.d. 11.1) vs CG: 38.8 (s.d. 11.1). Mean difference −0.78 (−2.58 to 1.03) p = 0.39. See individual columns for other outcomes.	No difference	No difference	No difference	Not reported	No difference	No difference	No difference	Not reported	IG: 15 (3.4%), CG: 14 (3.1%)
Murphy et al., 2013, Australia	Two year risk of a recurrent cardiac event using the Framingham algorithm for men and women with established CVD	2-year risk of CVD %: 4 months: IG: 8.46% (sd 3.12) vs CG 8.24% (sd 3.32); F = 2.54 (p = 0.056). 1 year: IG 8.43% (sd 3.18) vs CG 8.12 (sd 3.39), F = 0.84 (p = 0.179).	Not reported	No difference	Favours intervention	Not reported	No difference	Not reported	Not reported	Not reported	Not reported
Otterstad et al., The Vestfold Heartcare Study Group, 2003, Norway	Five-year risk of CHD (%) (non-fatal MI and combined fatal CHD) estimated using the WOSCOPS study algorithm (which is only applicable for males)	5-year CHD risk reduction: RRR baseline—6 months: IG: 22.6% vs CG: 6.1%, Mean difference: 16.5 (5.9–27.2) p<0.001. RRR baseline- 2 years: IG: 21.7% vs CG: 0.9%, Mean difference: 20.7 (7.8–33.7), p<0.001.	Favours intervention	Favours intervention	Favours intervention	Not reported	Not reported	No difference	No difference	Not reported	IG: 2 (2%), CG: 1 (1%)
Varnfield et al., 2014, Australia	Update, completion and adherence to CR programmes.	Uptake: IG: 80%, CG: 62%, p<0.05, completion: IG: 80%, CG: 47%, p<0.05, adherence: IG: 94%, CG: 78%, p<0.05.	Not reported	No difference	No difference	Not reported	Not reported	No difference	Favours intervention (for DPB)	Not reported	Not reported
West et al., 2012, England and Wales	The primary endpoint was mortality at 2 years	Total deaths: IG: 82 vs CG: 84,RR 0.98 (95% CI 0.74 to 1.30).	No difference	Favours control	No difference	Not reported	Not reported	Not reported	Not reported	No difference	Total deaths: 12 months: IG: 54 (6%) vs CG 47 (5.2%), RR 1.16 (95% CI 0.79 to 1.69). 2 years: IG: 82 vs CG: 84,RR 0.98 (95% CI 0.74 to 1.30). 7–9 years: IG: 245 vs CG: 243, RR 0.99 (95% CI 0.85 to 1.15)
Wister et al., 2007, Canada	The primary outcome was the global cardiovascular risk score—the Framingham risk-scoring method, which combines smoking status, total and high-density lipoprotein cholesterol, systolic blood pressure and fasting glucose level.	Framingham risk score: 1 year: IG: 6.75 (5.88–7.62), 5.9% change vs CG: 8.11 (7.25–8.97), 3.0% change. F = 0.13, p = 0.71.	No difference	No difference	No difference	Not reported	No difference	No difference	No difference	Not reported	Not reported
Yan et al., 2014, China	Illness perceptions assessed by the Chinese version of the revised Illness Perception Questionnaire	Identity: (IG: 5.94, CG: 3.84, p<0.001), Timeline (acute/chronic): (IG: 2.80, CG: 3.54, p<0.001), Timeline (cyclical): (IG: 3.11, CG: 3.09, p>0.05), Consequences: (IG: 3.54, CG: 3.78, p>0.05), Personal control: (IG: 3.82, CG: 3.16, p>0.05), Treatment control: (IG: 3.86, CG: 3.68, p>0.05).	No difference	Favours intervention	No difference	Not reported	Not reported	Not reported	Not reported	Not reported	Not reported
Zhao et al., 2008, China	Main outcomes were adherence to diet, medications, exercise and health related lifestyle and health care utilisation	Number with "high" adherence to activity: 12 weeks: IG: 90% vs CG: 62% (p<0.00). Number with "high" adherence to diet: 12 weeks: IG: 50% vs CG: 33% (p<0.05). Number with "high" adherence to medication: 12 weeks: IG: 86% vs CG:51% (p<0.00)	Not reported	Favours intervention	Favours intervention	Favours intervention	Not reported	Not reported	Not reported	Not reported	Not reported

#### Health behaviours

**Physical activity:** Although physical activity was assessed by 20 studies as an outcome, it was measured in a number of different ways and the data could not be combined statistically. It was, for example, assessed using a physical activity questionnaire, or as the proportion of the sample who met a specified criteria, e.g. exercising 5 times per week. Twelve of the 20 studies reported a statistically significant finding, indicating a positive impact of the intervention on physical activity.

**Diet:** Fifteen of the studies measured diet as an outcome, but the definitions were very different; from a Mediterranean diet score, to servings of fruit and vegetables, to dietary fat intake. Nine of the 15 studies reported a statistically significant improvement in diet in the intervention compared to the control group.

**Fig 3:**
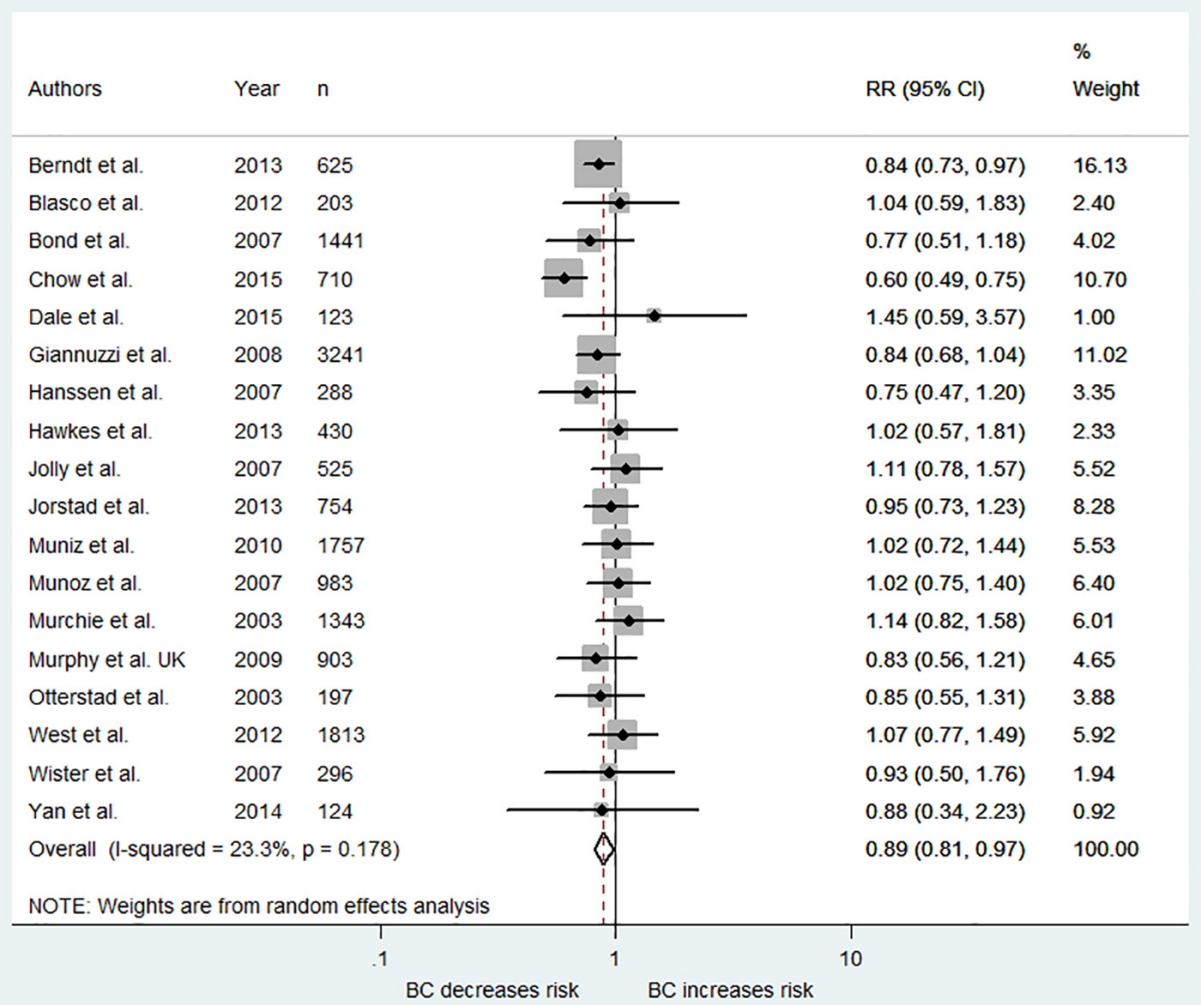
Forest plot showing weighted risk ratio for smoking.

**Smoking:** Fifteen studies reported on prevalence of smoking at follow-up and all of these were included in the meta-analysis (see Fig 3). The weighted risk ratio across these studies indicated a small but significant effect size (RR = 0.89, 95% CI 0.81–0.97), indicating that overall the interventions were more likely to result in smoking cessation compared to the control arms. Random effects models indicated that there was low heterogeneity between studies (*I*^*2*^ = 23%).

**Medication adherence:** Nine studies reported on medication adherence to cardioprotective medications [19, 23–25, 27, 31, 33, 34, 38] with four of these assessing self-reported medication adherence [19, 33, 34, 38]. Three of these studies reported that the intervention had a positive impact on adherence [27, 34, 38].

#### Intermediate outcomes

**BMI:** Data was available for 8 studies, for both the intervention and control arms, on mean BMI at follow-up. The weighted mean difference between the intervention and control groups at follow-up was -0.39 kg/m^2^ (95% CI -1.03–0.25) suggesting that there was not a significant difference between the groups for BMI. There was high heterogeneity across studies (*I*^*2*^ = 89%).

**Blood pressure:** Data was available for 10 studies for means at follow-up for systolic blood pressure (SBP) and for 9 studies for diastolic blood pressure (DBP). The weighted mean difference for SBP suggested a significant difference between the intervention and control groups of -3.13 mmHg (95% CI -5.11 - -1.15) with moderate heterogeneity across studies (*I*^*2*^ = 69%). There was a small significant difference between the groups for DBP with a weighted mean difference of -1.12 mmHg (95% CI -2.10 –-0.13) and moderate heterogeneity across studies (*I*^*2*^ = 53%).

#### CHD events and mortality

**Fig 4:**
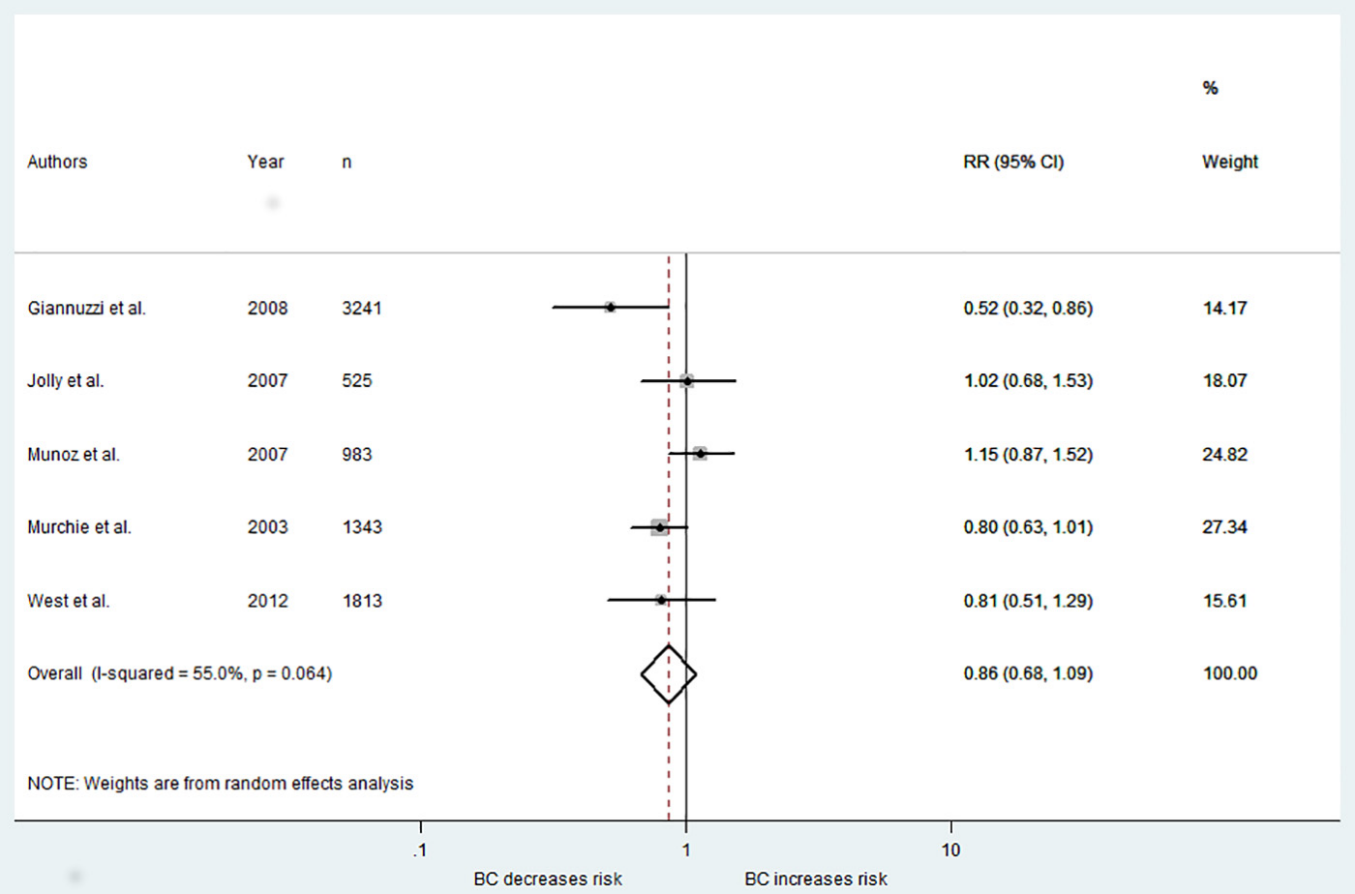
Forest plot showing weighted risk ratio for CHD events.

Five studies reported on the risk of CHD events between baseline and the final follow-up after the intervention. Four of these reported on the number of myocardial infarctions or total non-fatal cardiac events [8, 20, 23, 26], whereas one study reported only on the combined number of coronary deaths and non-fatal myocardial infarction events [27]. The weighted risk ratio indicated that the interventions did not have a significant effect in reducing the risk of CHD events (RR = 0.86, 95% CI 0.68, 1.09) and there was moderate heterogeneity across studies (*I*^*2*^ = 55%) (see Fig 4).

**Fig 5:**
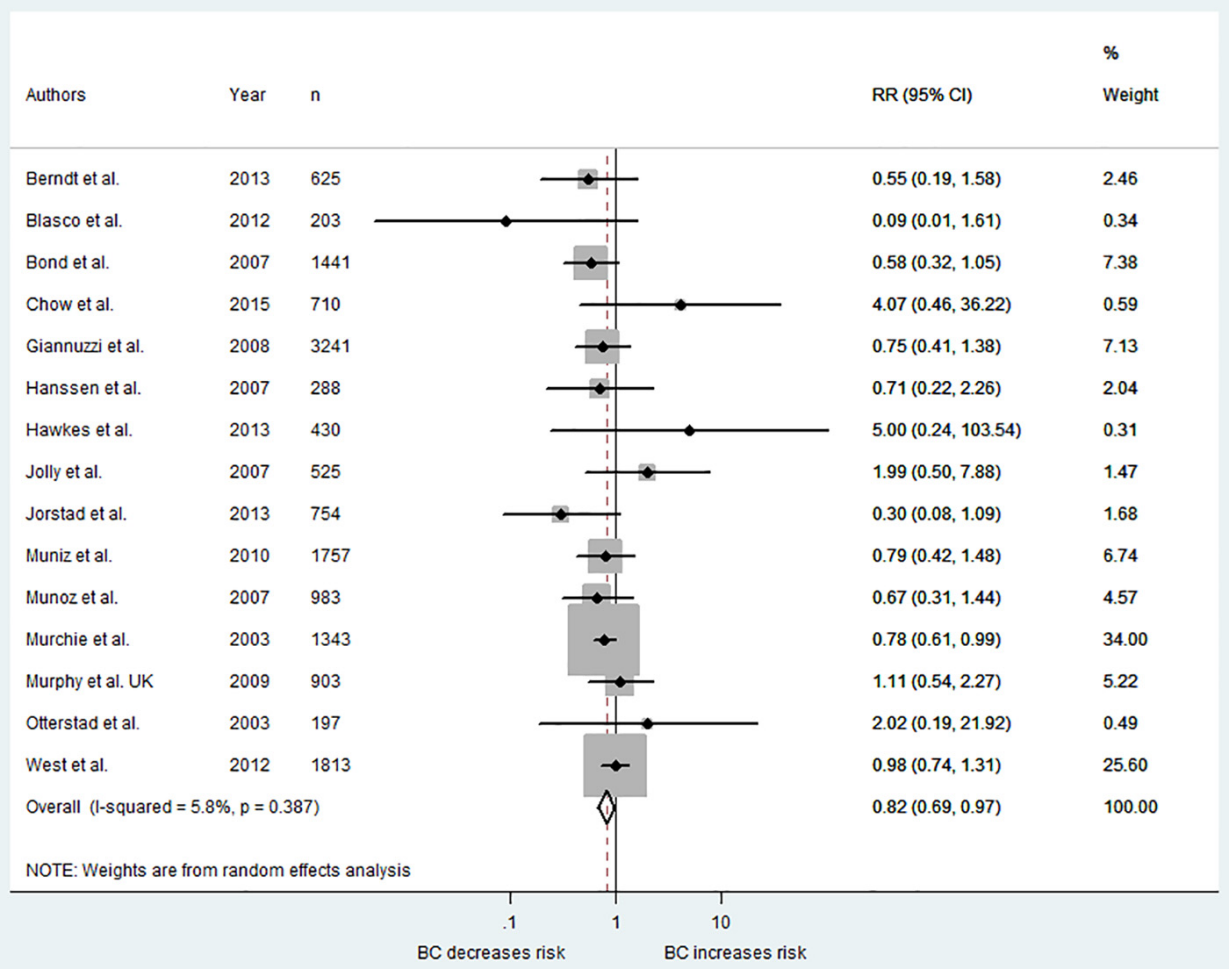
Forest plot showing weighted risk ratio for mortality.

The total number of deaths was reported in 15 studies, even though for many it was not reported as an outcome and was only referred to in relation to the flow of participants. The weighted effect across all 15 studies indicated a small significant effect size of the interventions in reducing risk (RR = 0.82, 95% CI 0.69, 0.97), with low heterogeneity across studies (*I*^*2*^ = 6%) (see Fig 5).

### Predictors of effects

#### Intervention length

The 15 studies which reported on mortality were stratified by length, suggesting that the longer interventions may have better outcomes; less than 3 months (RR = 0.97, 95% CI 0.75–1.26), 3–6 months (RR = 0.79, 95% CI 0.37–1.66), and 12 months or longer (RR = 0.76, 95% CI 0.62–0.92) although the difference in estimates was not statistically significant. The same pattern was not shown for the length of the intervention on risk of smoking; less than 3 months (RR = 1.06, 95% CI 0.87–1.30), 3–6 months (RR = 0.81, 95% CI 0.70–0.95), and 12 months or longer (RR = 0.92, 95% CI 0.80–1.04).

#### Individual or group delivered

The interventions were stratified by whether they were delivered on an individual basis or in a group setting, with the results for mortality suggesting that the individual interventions (RR = 0.76, 95% CI 0.61–0.95) may be more effective than the group interventions (RR = 0.94, 95% CI 0.73–1.22) although this difference was not statistically significant. The same analyses with smoking as the outcome showed no difference between these groupings; individual (RR = 0.89, 95% CI 0.80–1.00) and group interventions (RR = 0.89, 95% CI 0.76–1.05).

#### Theoretical basis

Studies were stratified on the basis of whether the intervention had been developed based upon psychological theory. The results for mortality suggested that the interventions without a theoretical basis (RR = 0.79, 95% CI 0.67–0.94) were more effective than those with a theoretical basis reported (RR = 1.06, 95% CI 0.65–1.75), but the difference between these groups was not statistically significant. The results for smoking did not suggest that there was a difference between these groups: no theoretical basis (RR = 0.95, 95% CI 0.85–1.05) and theoretical basis (RR = 0.83, 95% CI 0.70–0.99).

#### Inclusion of BCTs

Sensitivity analyses were conducted examining the different categories of BCTs as predictors of mortality in meta-regressions, in addition to a model examining the number of BCTs. The analyses did not show that any of the variables predicted mortality: goal setting/action planning (β = -0.06, 95% CI -0.28–0.15), review of goals/self-monitoring (β = -0.07, 95% CI -0.25–0.11), stress management (β = 0.08, 95% CI -0.13–0.30), social support (β = -0.06, 95% CI -0.25–0.14) and providing feedback (β = -0.03, 95% CI -0.20–0.15). The number of BCTs included in an intervention was also not associated with mortality (β = -0.02, 95% CI -0.06–0.03).

### Quality assessment

**Fig 6:**
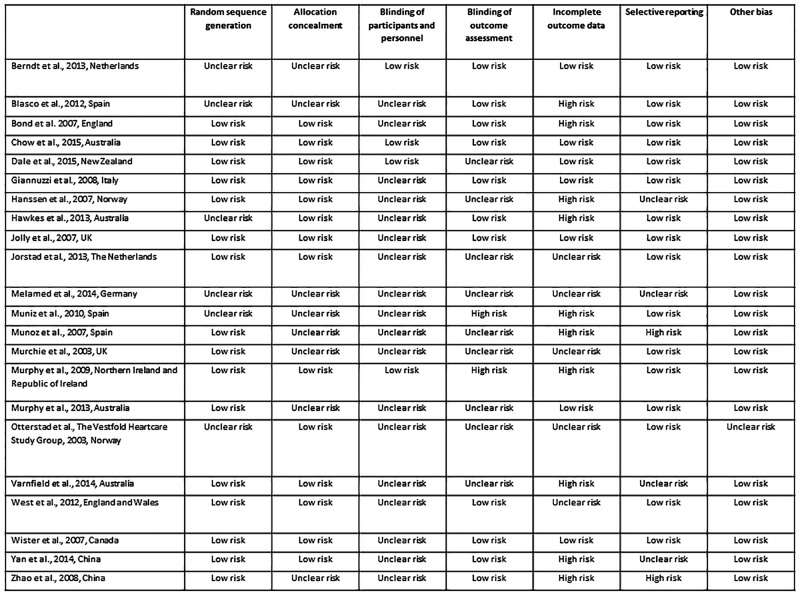
Risk of bias summary for all individual items.

The risk of bias for every domain for each study is reported in Fig 6. The majority of studies had a “low risk” for the selection bias domain, which includes random sequence generation and allocation concealment. Blinding was generally not possible for both patients and those who administered the intervention. For this reason most of the studies were considered to have an “unclear risk”. Attrition bias was one of the main issues in terms of quality. For almost half of the included studies the risk of attrition bias was considered to be “high”, as incomplete outcome data were not adequately addressed. The high prevalence of “unclear risk” judgments reflects the lack of clear reporting rather than a clear evidence of bias. This is in line with the finding of a general sub-optimal reporting of RCTs despite the large diffusion of instruments designed to help transparent reporting, such as the CONSORT Statement [39].

## Discussion

This review identified twenty two behaviour change intervention studies, from a range of countries, evidencing an overall positive impact of these interventions on smoking, systolic and diastolic blood pressure and mortality, but no effect on BMI or CHD events. There was limited evidence that longer interventions may be more effective in reducing mortality risk than those lasting for three months or less, and that interventions delivered on an individual basis (rather than as a group) may also be more likely to reduce the risk or mortality. Although this review aimed to identify which BCTs may be most effective in CHD patients, there was no association between individual BCTs with mortality, nor with the number of techniques used in an intervention.

This review was unique in extracting data on a range of outcomes. However, there was such heterogeneity in the measurement of health behaviours, such as diet and physical activity, that it was not possible to combine the results across trials in meta-analyses. The trials did seem to be effective in reducing the risk of smoking, albeit only a small decrease in risk, even though all but one were not designed specifically as smoking cessation programmes. This is in agreement with existing findings that smoking cessation programmes are effective in CHD patients [40]. Although secondary prevention guidelines for CHD tend to focus on pharmacological management of risk factors (e.g. UK Quality and Outcomes Framework; [41]), only nine studies reported on medication adherence as an outcome, and only three of these reported a positive outcome. For many participants, adhering to medication may be a more tangible change than modifying his/her diet, or increasing frequency of exercise, so this may have been a missed opportunity in trials which did not target adherence. Even though there were only small changes in the intermediate risk factors of blood pressure and BMI, there was still a positive effect of the trials on the risk of mortality. The effect size for mortality was also in line with previous reviews of psychological interventions for CHD (e.g. [13]).

The different studies varied in how the primary outcome was defined, most likely as a result of the range of outcomes available to select in this type of trial. Although a number of studies tackled this choice by using a combined outcome, such as the Framingham algorithm for CHD risk, an equal number stated multiple primary outcomes which the CONSORT guidelines advise against [42]. Other studies reported the primary outcome as meeting clinical guidelines for a range of risk factors based upon a score or algorithm [24, 29–32], but this was also open to misinterpretation if the primary outcome was stated to be met if a single guideline was achieved [19]. One of the implications of this review is that complex interventions targeting multiple outcomes may still have to select a single outcome (such as medication adherence) as the primary outcome, as long as the other constituent outcomes are reported in detail as secondary outcomes.

One of the aims of this review was to use an existing taxonomy to code behaviour change interventions, and to identify which techniques may be most helpful to this patient group. Whilst we were able to code all of the interventions and examine which were the most commonly used techniques, such as providing information and goal setting, we did not find evidence that it was these or other techniques which contributed to the overall effectiveness of the interventions. This may be due to the fact that most interventions included a range of techniques and there may be a synergistic effect of combining different techniques, as opposed to single techniques working alone. One example of this was that goal setting was included more commonly than review of outcome goals, even though these strategies would be expected to be implemented together. There has also been debate as to whether including more or less BCTs may have the most positive outcomes; an intervention with fewer BCTs may be more coherent and therefore easier to ensure intervention fidelity [43]. Coding of inclusion of BCTs was also only based upon the written information provided in the papers, which may not have been complete descriptions.

A limitation of the current behaviour change taxonomies is that they do not provide detail on specifically how the BCTs were applied (i.e. the clinical competence attached to the technique) and additionally how long each technique was used for, which could be the crucial element in determining efficacy. For example, on paper, goal setting appears to be a simple technique which was used commonly across the interventions, and that is also evaluated favourably by clinicians (e.g. [44]). However, it may still be open to misuse and could have been implemented quite differently, e.g. if the goals were set by clinicians rather than patients.

The interventions in this review were most commonly delivered by nurses. In some studies this was specific cardiac nurses, but some studies did not seem to prioritise this illness specific experience. The recent competence framework, specific to psychological interventions for people with physical health conditions, highlights 7 domains including generic therapeutic competencies for psychological interventions and condition specific interventions [45]. This framework suggests that whilst there are psychological techniques that can be applied across any patient group, a level of expertise is required that is specific to a condition. Further issues such as disease specific training and supervision are also highlighted in this framework [45], which were not summarized in many of the studies included in this review.

### Strengths and limitations

The strengths of this review are that it involved a comprehensive data extraction of a range of outcomes for CHD interventions, developing previous reviews both in the detail of the extraction and in undertaking BCT coding. One of the potential limitations is that there may not have been sufficient statistical power to examine the individual BCTs in meta-regression as predictors of outcome; however, the size of the effects found did not indicate that the null result was a consequence of lack of power. It was also not possible for us to assess publication bias, and methods such as assessing asymmetry in funnel plots are not recommended when there are a small number of studies [46]. Many of the studies included were assessed as having a high risk of attrition bias, which could have affected the preciseness of the estimates in the meta-analyses. The risk of bias assessment did not suggest that there was likely to be reporting bias due to selective reporting of outcomes.

There was considerable variation between studies in the measurement and definition of the health behaviour and biological outcomes. The majority of the health behaviour outcomes were also self-reported and there is potential that social desirability effects may have been greater if participants knew they were in the intervention group. This review applied broad inclusion criteria and patients who had experienced different treatment were pooled together. However, this was consistent with a pragmatic approach aimed at emphasising the generalisability of these results to healthcare settings more widely.

### Clinical and research implications

One of the implications from this review for clinical practice relates to whether healthcare commissioners should be investing in new psychological behaviour change interventions, or if existing cardiac rehabilitation programmes could be further developed both in the content and through tackling issues which have led to poor uptake and adherence. Although the West study showed no positive effect of CR in a multi-centre RCT, other studies have argued that this may have been due to lack of power to detect change due to the trial terminating early [47] and a further Cochrane review of CR did find a positive effect on mortality [48]. Many of the studies in this review compared their intervention to usual care which included CR and showed positive outcomes, but in the main these effects were small. A number of the more recent interventions used automated text messaging for delivery [19, 33–35]. These methods seemed to be as effective as the face-to-face programmes, but are more likely to be cost effective and easily implemented on a wider scale.

We have identified a number of generic difficulties in reporting and synthesising intervention studies in this area, which should be addressed in future research. *1) The content of the intervention and the method of delivery were not clearly reported*. The BCT taxonomies are helpful in defining the intervention techniques, but this will be most useful when this information is embedded within the content of the intervention (e.g. goals were set in relation to gradual increases in activity versus smoking cessation) and reports on how the techniques were delivered (e.g. using guided discovery and collaborative decision making versus a more didactic approach). Making treatment manuals publically available will enable better comparison of studies. *2) There was high heterogeneity and lack of specificity in the definition of ‘treatment as usual’*. This issue may to some extent be unavoidable due to different practices in cardiac rehabilitation across regions and countries, but it can be improved through applying greater rigour and standardisation in the coding of treatment received by the control group. In some studies it was stated that control participants could be referred to cardiac rehabilitation, but whether referral/attendance actually occurred was not always taken into account in the evaluation. The use of BCT coding for the control treatment may additionally be helpful. *3) There were reporting issues for the primary outcome measure*. In this area of research multiple outcomes are relevant and one health behaviour or physiological outcomes cannot necessarily be prioritised over another. Whilst CHD events or mortality may be an obvious primary outcome, many pilot or early stage intervention studies will not be powered to detect these outcomes. Reporting multiple endpoints may be the most appropriate option, even though this is problematic statistically, it would not fit with current reporting frameworks and there is scope for reporting bias. Furthermore there are some behavioural outcomes, for example smoking, which are not undertaken by all participants in a trial so additional reporting guidelines are required on the most appropriate evaluation approach.

### Conclusions

Although this review found evidence for a positive effect of these secondary prevention interventions on smoking, systolic blood pressure and mortality, the major challenge is in defining the content of the interventions and identifying what might be the active components. Using a BCT taxonomy helped us to understand which were the most commonly used techniques, providing information and goal setting, but it was not possible to identify what were the essential ingredients of these behaviour change interventions.

## Supporting Information

S1 Table PRISMA checklist (DOCX)
